# Is this really Empowerment? Enhancing our understanding of empowerment in patient and public involvement within clinical research

**DOI:** 10.1186/s12874-024-02323-1

**Published:** 2024-09-13

**Authors:** Imke Schilling, Ansgar Gerhardus

**Affiliations:** 1 Department for Health Services Research, Institute of Public Health and Nursing Research, University of Bremen, Grazer Straße 4, 28359 Bremen, Germany; 2 Health Sciences Bremen, University of Bremen, 28359 Bremen, Germany

**Keywords:** Patient and public involvement, PPI, Empowerment, Clinical Research

## Abstract

**Background:**

There has been a growing push to involve patients in clinical research, shifting from conducting research on, about, or for them to conducting it with them. Two arguments advocate for this approach, known as Patient and Public Involvement (PPI): to improve research quality, appropriateness, relevance, and credibility by including patients’ diverse perspectives, and to use PPI to empower patients and democratize research for more equity in research and healthcare. However, while empowerment is a core objective, it is often not clear what is meant by empowerment in the context of PPI in clinical research. This vacancy can lead to insecurities for both patients and researchers and a disconnect between the rhetoric of empowerment in PPI and the reality of its practice in clinical trials. Thus, clarifying the understanding of empowerment within PPI in clinical research is essential to ensure that involvement does not become tokenistic and depletes patients’ capacity to advocate for their rights and needs.

**Methods:**

We explored the historical roots of empowerment, primarily emerging from mid-20th century social movements like feminism and civil rights and reflected the conceptual roots of empowerment from diverse fields to better understand the (potential) role of empowerment in PPI in clinical research including its possibilities and limitations.

**Results:**

Common themes of empowerment in PPI and other fields are participation, challenging power structures, valuing diverse perspectives, and promoting collaboration. On the other hand, themes such as contextual differences in the empowerment objectives, the relationship between empowerment and scientific demands, research expertise, and power asymmetries mark a clear distinction from empowerment in other fields.

**Conclusion:**

PPI offers potential for patient empowerment in clinical trials, even when its primary goal may be research quality. Elements like participation, sharing opinions, and active engagement can contribute to patient empowerment. Nonetheless, some expectations tied to empowerment might not be met within the constraints of clinical research. To empower patients, stakeholders must be explicit about what empowerment means in their research, engage in transparent communication about its realistic scope, and continuously reflect on how empowerment can be fostered and sustained within the research process.

## Background and problem statemen

### Introduction

There has been a growing demand from patients, researchers, research sponsors, and scientific journals to shift clinical studies from being exclusively conducted on, about, or for patients to involving patients themselves or members of the public [1, 2]. Two primary lines of reasoning underlie active patient and public involvement (PPI):

1. By integrating patients’ diverse perspectives into research, the aim is to enhance the quality, appropriateness, relevance, and credibility of the research [3, 4].
2. Additionally, there are normative arguments supporting PPI that revolve around moral, ethical, and rights-based considerations, primarily linked to empowering patients or the public [5]. In essence, the idea is that patients should have a say in research that directly concerns them [3, 6]. This notion aligns with the principle of “nothing about us, without us,” which has guided movements in various contexts, including the disability rights movement [7] and Indigenous contexts [8].

By empowering patients and upholding their right to participate in research, PPI seeks to diminish social inequalities. In doing so, it aims to democratize the research process, making it more accountable and transparent to the broader population [2–5, 9]. This democratization is particularly significant for marginalized groups whose perspectives are often overlooked [1, 5].

While patient empowerment is a core objective of PPI [4], it is seldom explicitly defined within the context of PPI. Based on the etymology, the root of the term implies that ‘empowerment’ concerns matters of ‘power’. The Oxford English Dictionary offers three distinct meanings of the verb “empower” [10]. One involves granting someone legal or formal authority, another focuses on bestowing power over something, and the third pertains to strengthening an individual by providing greater control, specific attributes, or enhanced abilities. Empowerment can denote either a process or a state of being respectively an outcome.

A narrative review by Gradinger et al. revealed that in the context of public involvement, normative values are frequently referenced without clear definitions, resulting in significant variations in the understanding of empowerment [4]. While there is a general need to clarify the conceptualization of PPI to align with its intended goals [11], the emancipatory aspect of PPI remains underexplored compared to other approaches [12]. Without a precise meaning and operationalization of the term ‘empowerment’, the normative claim of PPI becomes difficult to realize and its implementation virtually impossible to assess. The lack of a shared understanding of empowerment within PPI not only fosters misinterpretation and arbitrariness in PPI practices but may also inadvertently undermine patient empowerment. From the perspective of a patient, ambiguous roles, a sense of inability to contribute, insufficient recognition of one’s contributions, or inadequate information about the benefits of involvement could potentially be rather disempowering than empowering [13]. There is a risk that the involvement may become tokenistic, and patients’ voices might be silenced when they are merely involved for show, as a formality, without genuine influence on the research. Additionally, this involvement may deplete patients’ resources and capacity to advocate for their rights and needs in potentially more effective ways [14, 15].

In a previous study, we discovered that within the same project, patients and researchers assign varying degrees of importance to patient empowerment. While patients engaged in a patient board for a clinical trial endorsed the idea of empowerment through research participation, only one out of five researchers explicitly addressed patient empowerment as a rationale for conducting PPI [16]. Furthermore, the experiences of patients and researchers with the patient board indicated that patient empowerment is often overlooked in the implementation of PPI. Other forms of collaboration, such as open dialogues on an equal footing and providing training to enhance patients’ confidence and skills, might have proven more effective in empowering patients [17]. These findings align with those of Ives et al. [3], who also noted a potential mismatch between the stated goals of PPI and its practical execution. Ives et al. argue that the nature and conduct of PPI can vary significantly depending on who initiates it and for what purpose. For instance, if researchers involve patients primarily to enhance the quality of their research projects, the focus might be on outcome-oriented, pragmatic consultation, potentially sidelining the goal of patient empowerment. Patients may be relegated to an informational role rather than active partners in the research process. Based on these insights, we assume that empowerment does not naturally evolve from PPI and is not an automatic byproduct of it.

### Aim, research interest and approach

Considering the above, it seems necessary to clarify the term empowerment within PPI in clinical research. Despite the absence of a precise understanding of empowerment in the context of PPI, the term “empowerment” has been in use across various domains for over half a century, including social work, education, corporate settings, psychology, and healthcare [18]. Therefore, this article aims to contribute to the understanding of empowerment in PPI by reflecting on the history and tradition of the term and concept of empowerment in other fields. Building on this, we aim to reflect on what lies behind the term empowerment in the context of PPI in clinical research and try to explain the disconnect between the rhetoric of empowerment in PPI and the reality of its practice in clinical trials. We have been guided by the following questions and have structured the article accordingly:

- How has the concept of empowerment evolved historically?
- How has empowerment been conceptualized in other fields?
- To what extent does the concept of empowerment of patients through or for PPI in clinical research align with conceptual approaches to empowerment in other fields?

The article provides researchers who organize PPI with orientation on the relationship between empowerment and PPI. It offers perspectives on the possibilities and limits of empowerment in this context and invites further reflection on the topic from both researchers and patients involved in PPI.

For consistency, the term ‘patient’ is exclusively used in this article to refer to individuals who have had specific health-related experiences. However, we acknowledge that other terms, such as ‘service users’, may be more suitable and better reflect the active role that PPI strives for. This article is centered around PPI in clinical research and does not encompass reflections on PPI in other contexts, such as healthcare.

## Historical development of the term empowerment

The term “to empower” has been documented since the mid-17th century, with older forms such as ‘impover,’ ‘empour,’ and ‘empowre’ [10]. In the mid-17th century, William Penn, founder of the Quaker colony of Pennsylvania, utilized the term in a religious and early democratic context. Penn’s theology of individual empowerment was based on the belief in the intrinsic dignity of all individuals, the presence of a part of God within each person (referred to as the “inward light” or “inner spirit”), and the assertion of the right to freedom of conscience. Penn’s ideas influenced the formulation of a groundbreaking constitution for Pennsylvania, serving as a model for subsequent democratic constitutions [19].

### History of empowerment in the social movements

The term “empowerment,” intertwined with democracy since its inception, has evolved over time, primarily shaped by mid-20th-century social movements.

#### Civil Rights Movement and Black Empowerment

The civil rights movement in the 1950s and 1960s among the Black minority in the U.S. significantly influenced the idea and implementation of empowerment. Acts of civil disobedience exposed racial inequalities [20], and multiplier programs aimed to provide education and raise consciousness among the Black community [18, 20]. Grounded in the belief in individuals’ abilities to control their lives, the movement sought to integrate the Black minority as equals with equal social rights into the democratic society. Freeing the Black minority community from oppression through collective self-organization resulted in a “new sense of somebodiness” (Martin Luther King as cited in Simon [19]).

#### Feminist movement

Another driver of the empowerment discourse was the second wave of the feminist movement in the 1960s and 1970s, which addressed women’s opportunities and rights for societal equality [21]. Through expanded education, improved labor conditions, economic independence, far-reaching changes in the possibilities for self-determined birth control and a developed awareness of personal (bodily) autonomy, women’s life plans became more individualized [22]. Within the movement, women found a protective framework to navigate their evolving opportunities and resulting responsibilities. It provided a social reference structure, creating spaces for self-clarification, collective articulation of devaluation, and deconstruction of internalized beliefs. This support allowed women to envision, develop, and test new life possibilities and identities, thereby fostering self-confidence [18].

#### Self-Help movement

A third root of the modern empowerment concept is the self-help movement, which gained importance in the 1970s in the USA and other developed countries, especially within health-related contexts [7, 18]. As self-organized networks, self-help aimed to establish social support, explore coping strategies, and reclaim autonomy and empowerment resources. Self-help served as a counter-program to perceived disempowering state care [7], emphasizing the perspective of individuals as ‘experts on their own account’, introducing self-organized services, creating (a sense of) community and thus producing emotional ‘services’, empowering critical consumers, and representing peoples’ interests to influence socio-political decisions [18]. Key features of self-help networks included the involvement of members with a common problem, minimal professional helper involvement, emphasis on immaterial support, and goals of self- and social change achieved through equal cooperation and mutual help. Self-help groups provided critical support in niches not covered by professional care services [18].

#### Community action programs and community psychology

In the U.S., community-based programs aimed at empowering individuals and building networks to address social segregation [23]. These programs furnished resources and support to enable individuals and communities to take charge of their lives and implement positive changes in their community. Political initiatives, like the Equal Opportunities Act of 1965, sought to reduce inequalities and poverty, promoting “maximum feasible participation” [18]. Empowerment was considered a means of encouraging self-sufficiency and reducing dependence on government support.

In the 1970s, community action programs became linked to community psychology, viewing individuals as part of communities and collaborating to identify strengths, resources, and needs. Strategies formulated aimed to empower and promote social justice while reducing social inequalities.

The tradition of empowerment in social movements encompasses both individual self-determination and collective action against structural constraints. The primary concerns were not only about self-empowerment but also about advocating for structural changes through mass mobilization and collective efforts. In these contexts, empowerment was often pursued through independently organized groups that fostered community solidarity and collective identity. Unlike prevalent deficit-based approaches, which tend to focus on individuals’ lacks and weaknesses, empowerment in social movements nurtures and strengthens individuals’ skills and capabilities while also addressing and dismantling oppressive structures.

### Today: use in various contexts

Since its emergence in mid-20th-century social movements and subsequent development in community psychology, the concept of empowerment has found application across diverse domains [18, 24]:

- Social work, encompassing individual support and collective actions.
- Educational programs, such as literacy campaigns and increased pupil participation opportunities.
- Development aid, representing a shift from external, top-down approaches to fostering local community capacity for participatory development and poverty reduction in developing countries.
- Corporate contexts, where empowerment principles are integrated into management strategies.
- Healthcare, where applications include shared decision-making and broader patient involvement.
- Contemporary movements, such as racial empowerment in the “Black Lives Matter” movement and the Indigenization.

## Concepts of empowerment

In this section, we explore the foundational concepts and theoretical underpinnings of empowerment.

### Key concepts related to empowerment

#### Solomon

Social scientist Barbara Bryant Solomon pioneered the conceptual foundation of empowerment in her 1976 book, “Black Empowerment: Social Work in Oppressed Communities.” Originating as a resource for students and social workers assisting Black minority clients, Solomon’s empowerment concept is based on research into the mechanisms of power and powerlessness. According to her, “empowerment refers to the reduction of an overriding sense of powerlessness to direct one’s life in the direction of meaningful personal satisfaction” [25]. At the core of this concept is the experience of powerlessness, arising from membership in a minority group subject to negative assumptions and discrimination from the majority society and its institutions [26, 27].

While previous authors had emphasized the need to consider stigma as a factor that permeates the social situation of Black people, Solomon added that the unequal distribution of power and the experience of (structural) discrimination could affect the psyche and the negative attributions could find their way into self-perception. Thus, powerlessness of an individual means “the inability to manage emotions, knowledge, skills or material resources in a way that makes possible effective performance of valued social roles so as to receive personal gratification” [26].

At the community level, powerlessness is described as the inability to utilize resources for collective goals [26]. In short, stigma affects powerlessness, hindering access to the resources necessary for overcoming negative self-perceptions and social challenges [27]. Introducing empowerment as a method, Solomon suggested that professionals could employ it to address the powerlessness experienced by stigmatized individuals or groups. Empowerment, in her view, enables individuals to recognize their competence, perceive available opportunities for control, and ultimately enhance their self-worth and dignity [25]. In summary, Solomon’s empowerment approach is based on the belief that individuals and families have strengths and abilities and that they can be supported to use their resources more effectively for their own benefit. Solomon saw empowerment as both a process and a goal for social work in Black communities, and stated that the success of empowerment is “directly related to the degree to which the service delivery system itself is an obstacle course or an opportunity system” [26].

#### Rappaport

In 1981, community psychologist Julian Rappaport advocated for empowerment as a superior approach to paternalistic public health policies and rights-based advocacy in social work [28]. Acknowledging the diverse nature of social problems, Rappaport urged professionals to reconsider their roles in relation to clients, aligning with Solomon’s view that empowerment enhances individuals’ control over their lives.

Rappaport emphasized viewing individuals not solely as children in need or rights-bearing citizens but as complete human beings with both rights and needs. He argued that even those seemingly incompetent and in need require “[…] more rather than less control over their own lives, and fostering more control does not necessarily mean ignoring them“ [28]. Increased control is believed to positively influence psychological well-being.

Empowerment, according to Rappaport, relies on the belief that people possess or can acquire competencies, with inadequate functioning attributed to social structures or the lack of resources that prevent people from using these competencies. He advocated for competency development in real-life settings and positioned those providing help as collaborative teammates who take into account social structures and living conditions, and not as authoritative experts [28].

Furthermore, Rappaport stressed the need for diverse solutions to divergent problems, rejecting a one-size-fits-all approach in social policy. He championed a bottom-up, participatory social policy that recognizes the context-specific and varied nature of empowerment in each situation [28].

#### Freire

Brazilian educator and social reformer Paulo Freire expanded the concept of empowerment through his work with marginalized communities in Brazil [29]. Central to his ideas is the development of ‘critical consciousness’ through dialogic education [30]. Freire contended that oppressed individuals often lack awareness of the social and political factors sustaining their subjugation. Critical consciousness involves recognizing oppressive systems and understanding the socio-economic and political contexts fostering inequality, along with realizing one’s potential for transformation. Freire regarded the critical consciousness experience as the key to gaining strength, with education playing a fundamental role to conscientization. Freire’s dialogic teaching method, emphasizing two-way learning between teachers and students, fosters critical thinking, self-reflection, and active participation, empowers students to question and reshape their reality. Working in partnership assigns the teacher the role of a facilitator and underscores the central importance of the consumer or marginalized individuals in the process of change [19, 30]. Complementing this, Freire’s pedagogy of questioning encourages students to critically assess the influences shaping their lives. The emphasis is not on remembering details, but on cultivating analytical skills and the capability to challenge prevailing beliefs.

Beyond individual liberation, Freire argued that true empowerment encompasses collective action and social transformation. He underscored the importance of solidarity and creating dialogic spaces for individuals to collaboratively address common experiences of oppression and work towards societal progress [30].

In summary, Freire sought to empower individuals and communities by promoting critical consciousness, dialogue, and collective action to challenge oppressive systems and foster a more inclusive and equitable society. While he placed responsibility on the oppressed for seeking their own empowerment, caution was advised to prevent reinforcing a sense of helplessness [29].

### Common principles

While there is no universally agreed upon definition or concept of empowerment, some common principles can be identified, then, from what we have reviewed: Empowerment comes from a variety of sources, refers to processes and outcomes, involves both personal and collective dimensions, is based on participation, assumes that each individual has strength and capacities upon which they can build, challenges power structures with a focus on marginalized groups and the systematic inequalities they face, and must be obtained by the individuals themselves, but can be supported by third parties, e.g. professionals, who facilitate the process of empowerment in collaboration with individuals or communities [19, 26, 28–31].

As the most basic definition of empowerment, Herringer outlines: “Developmental processes over time in which individuals acquire the skills necessary to live a life that meets their own standards of ‘better’” [32, translated by IS]. These processes of gaining more power or autonomy can be individual and collective [32].

### Controversies

At the same time there exist some controversies around empowerment. Herringer continues his definition with the thought: “[.] what exactly constitutes a “more livable” existence is open to conflicting interpretations and ideological frameworks” [32]. Other controversies surrounding the concept of empowerment are:

- *Instrumentalization*,* tokenism and depoliticization*: the concern that empowerment programs or initiatives may be implemented for instrumental, tokenistic purposes or to create the illusion of progress [27]. In such cases, empowerment becomes an empty concept without substantial impact. The adoption of empowerment concepts by the powerful (e.g. institutions or entities that hold significant structural and decision-making authority) can lead to a depoliticization of empowerment programs, as the transformative potential of such initiatives may be diminished or neutralized when circumscribed by institutional capture. This co-option of empowerment by those in power can result in a form of engagement that maintains existing power dynamics rather than challenging them.
- *Lack of clarity and measurement*: empowerment is so diverse and open-ended that it is difficult to define in a way that its outcomes can be measured [24]. Clarity is needed regarding which aspects of empowerment are targeted. Without evaluating empowerment attempts, it is challenging to learn from experience.

## Empowerment in the context of PPI

The concept of empowerment has deep roots in various social movements that sought to challenge systemic inequalities and give voice to marginalized groups. To analyze how these conceptual approaches to empowerment from social movements relate to the empowerment of patients in PPI within clinical research, we will first provide an overview of the historical development of PPI in research, followed by a recall of the relevance of empowerment in the context of PPI. We will then analyze and critically address (a) the similarities of approaches to empower patients or the public in PPI as compared with other fields, and by that get an impression how PPI in clinical research can empower patients, and (b) the distinctions and limitations of empowerment in this context, both practically and conceptually.

### Evolution of PPI in research

Patient advocacy movements, gaining momentum in the mid-20th century, played a pivotal role in pushing for increased patient involvement in research [33]. These movements, which often emerged from broader social and civil rights movements, laid the foundation for what we now recognize as PPI.

For instance, the HIV-AIDS activism of the 1980s, heavily influenced by the gay civil rights movement, led to significant changes in health research by challenging the prevalent research expertise and bringing in “a ´patient perspective` to bear on institutions of health research” [34].

In the 1970s, Rose Kushner, a breast cancer patient and writer, exemplified this movement by assessing research proposals for the US National Cancer Institute, marking a notable instance of patient influence [33]. Her efforts reflected a broader movement towards giving patients a voice in research, a theme that is echoed in many PPI initiatives. The 1980s collaboration between patient organizations and the Association for Maternity Services, endorsing a randomized controlled trial on chorionic villus sampling, is another example where patient involvement began to influence research decisions directly. The 1997 international breast cancer advocacy conference organized by the US National Breast Cancer Association (NBCC) and supported by patient organizations from several countries marked a pivotal shift towards PPI, fostering dialogue on patient experiences and challenges. The conference demonstrated the NBCC’s belief that breast cancer patients should be consulted when making policies and decisions regarding research funding, and was instrumental in establishing an international advocacy movement [35].

The connection between PPI and social movements became more explicit with the establishment of organizations like INVOLVE in 1996, funded by the British government as part of their aim to create a patient-oriented healthcare system, the Canadian Institutes for Health Research in 2000, and the Patient-Centered Outcomes Research Institute (PCORI) in the United States in 2010. These organizations, drawing inspiration from social movements, emphasize the importance of involving patients and the public throughout the research process, thereby continuing the advocacy for marginalized voices in health research [36–38].

Globally, there is a trend toward formalized PPI approaches. Research funders, regulatory bodies, and institutions recognize the importance of involving patients and the public throughout the research process, from prioritization to dissemination [1, 2]. At current there is still a lot of development and movement in the process.

### Relevance of empowerment in PPI

As discussed, there are two arguments advocating for PPI use in research, that Ives et al. summarize [3]: (1) to improve research quality, appropriateness, relevance, and credibility (PPI as a means to an end) and (2) to use PPI to empower patients and democratize research along with its consequential impact on health(care) (PPI as an end in itself). However, empowerment through PPI should not be seen as an isolated goal, and Ives et al. phrasing as “an end in itself” might be misleading and be better put as “an end beyond narrowly instrumental goals”. PPI is a strategy that allows patients to actively shape research, thereby ensuring that the research directly addresses the practical problems they face – an argument rooted in the social movements.

PPI is essential in transforming the relationship between patients and institutions, challenging traditional power dynamics [34]. Its role is dual-faceted: it improves the quality and relevance of research while simultaneously fostering a more participatory and inclusive approach to healthcare. This dual function makes PPI a powerful tool for achieving both immediate research goals and broader societal change.

However, depending on the reasons and initiators of PPI, PPI practices can vary greatly. According to Ives et al. [3], different aims of PPI can result in distinct forms of involvement, as illustrated in Table 1. While Ives et al. [3] seem to indicate two opposite ends of the spectrum, these “ideals” do not always play out and there are numerous intermediate forms of involvement that can exist. However, this example illustrates that the potential for empowerment in PPI, as well as its manifestations, can vary greatly depending on the approach taken.

**Table 1:** Aims and approaches of PPI [3]

	PPI as ‘means to an end’	PPI as ‘end in itself’
**Model**	Consultation by invitation	Partnership/alliance
**Approach**	• Top down • Pragmatic • Outcome oriented	• Bottom up • Rights based • Process oriented
**Purpose for research**	• Increases the relevance of research • Increases the quality of research (adds insight to the design, methods and findings; assists in dissemination and implementation)	• Representation of community values and preferences • Transparency and accountability • Equalising elitist and exclusionary power imbalances between the public and the academic community
**Nature of involvement**	• Information giving about decisions made • Invitation to respond	• Encourage new ideas and joint decision making
**Relationship**	Transactional	Cooperative

Today PPI spans a broad range, from sporadic consultations, to ongoing collaboration between patients and researchers, and even (still rare examples of) research led by patients with support from researchers [39].

### Similarity of empowerment in PPI in clinical research to earlier concepts

In the following sections we analyze and critically address the similarities and limits of empowerment in PPI in clinical research as compared with earlier concepts. Similarities of empowerment in PPI in clinical research to earlier concepts seem to be in a focus on participation, challenging power structures, valuing diverse knowledge and perspectives, and supporting collaboration.

#### Emphasis on participation

Active participation in decision-making processes that influence the lives of individuals and communities is a fundamental aspect of empowerment concepts across various fields [24]. In research-based PPI, facilitating the ability of patients and members of the public to have a voice, participate in decision-making processes, and contribute to research aligns with the core principles of empowerment.

#### Challenging power structures

Empowerment theories from different disciplines aim to reduce powerlessness and increase the power of marginalized individuals [25, 28, 29]. The objective of challenging power structures aligns with the concept of empowerment in PPI in research. Involving patients in planning, conducting, and communicating clinical research on a regular basis constitutes a significant shift in the power dynamics of the research landscape. Individual patients may be engaged on a one-time basis, but the collective voice of patients and the public becomes significant and co-determines research. Long-term patient involvement may be achieved through the integration of patient advisory boards in research institutions [40]. The inclusion of patient perspectives has become an expected practice, influencing power dynamics within the clinical research domain.

#### Recognition of diverse knowledge and perspectives

Empowerment in various fields recognizes the worth of diverse knowledge and perspectives [26, 28, 32]. By incorporating them, empowerment aims to challenge the conventional power structures that have systemically marginalized some voices and sustained inequality. Moreover, involving individuals with varied experiences offers exceptional insights and understandings that enhance dialogues and contribute to more thorough resolutions [28]. Similarly, patient experiential knowledge and unique insights are recognized as crucial in PPI for shaping research and complementing the specialist knowledge of clinical researchers [4, 13]. According to the Montreal Model, patients’ experiences with illnesses, which they must manage for the rest of their lives if chronically, offer a rich source of knowledge essential for decision-making [41]. This experiential knowledge includes patients’ insights into their health issues, the trajectory of their care, and the impacts on their personal lives and those of their loved ones [41]. The involvement of patients strengthens the focus of clinical research on patients’ needs, ultimately enhancing its quality, adequacy, relevance, and credibility [3, 4].

#### Collaborative relationship

Empowerment approaches typically foster collaborative relationships among various stakeholders [19, 28]. In social work, these relationships arise between the practitioner and the client and are characterized, analogous to the idea of an alliance, by a “shared sense of urgency” (regarding the client’s problems), a “conjoint commitment to problem solving in as democratic a manner as possible”, and a “shared emphasis [.] on [the] common humanity” in the relationship [19]. Depending on the PPI approach, the concept of collaborative relationships among various stakeholders can also apply to empowering patients in research. Three involvement approaches in PPI are distinguished [6]: (1) The consultation approach achieves the lowest level of engagement and collaborative relationships, wherein patients provide advice to researchers but are not involved in decision-making. (2) Patients are partners in the research process in the collaboration approach, with their involvement in decision making and shared responsibility for the research. (3) Patients in user-led research take full responsibility for individual aspects or the whole research, with support from researchers [6]. User-led research can only be implemented to a limited extent in clinical studies, as it is subject to ethical and legal framework conditions.

To strengthen the principles of social movements in PPI, a collective approach to research, as proposed by MacDonald’s theory of civic patienthood, could provide valuable insights [34]. This theory views patients as civic actors who seek collective solutions to collective problems, shifting the understanding of patients from merely clinical subjects to engaged participants in shaping research and healthcare outcomes. This approach needs robust institutions, resources, and socialization processes to support patients’ involvement. It is particularly critical in ensuring that PPI remains genuinely democratic and is not co-opted by more powerful interests [34].

### Distinctions of empowerment in PPI in clinical research to earlier concepts

While we found the heritage of social movements to inform the ethos of PPI in the principles of participation, giving people a say in decisions that affect their lives, confronting power structures—albeit on a smaller scale—, and collaborative relationships, we also found distinctions of empowerment in PPI in clinical research to earlier concepts. These seem to be in the areas of context and focus, scientific demands and ethics, expertise in research methods, and power dynamics.

#### Context and focus

While the goals of empowerment in other fields and PPI share similarities, there are differences in the context and focus. In social movements, empowerment refers to the process through which marginalized individuals and communities obtain power, active participation, and the ability to challenge oppressive systems [18, 29]. These movements often aim to effect systemic changes and combat inequalities, drawing upon collective action, raising awareness, and advocacy to achieve their goals [29, 30]. In contrast, the context of empowerment in PPI is more specific to the research process. Here, empowerment is about providing patients and the public with a voice in decision-making within that process [4]. While the influence of social movements is undeniable, the primary objective is not necessarily to address systemic inequalities on a broad scale but to enhance the quality and relevance of research by incorporating diverse perspectives. In PPI, people are empowered or given a voice “to influence research outcomes that will (or may) have a direct impact on their health status“ [6]. Though not the main objective, this involvement of diverse perspectives in research may nonetheless potentially contribute to a reduction in inequalities [42, 43].

However, the practical implementation of PPI often faces challenges that may undermine its empowering potential. Researchers, under pressure to demonstrate measurable impact, tend to focus the conduct of involvement on substantive values such as effectiveness, quality, and validity – outcomes that are more easily quantified and aligned with traditional research goals [4, 14]. This focus may lead to the marginalization of crucial but less easily measured normative values like empowerment, rights and accountability and process values such as partnership or respect. The demand for measurable outcomes and recommendations for the conduct of PPI that lead to rather structured and controlled PPI mechanisms shape PPI practices in ways that may suppress rather than amplify the voices of patients [14]. A more reflexive and dialogic approach to evaluating PPI might better capture its ethical and formative dimensions, ensuring that public involvement in research remains a tool for true empowerment rather than an instrument of containment [14].

#### Scientific demands and ethics

Empowerment in clinical research must balance patient empowerment with scientific demands and the integrity of research findings. Empowerment approaches in other fields may concentrate on personal growth and social change. However, in clinical research there is a need to find ways that respect both the methodological and ethical requirements of research and the interests of PPI. This aspect, which is specific to this context, distinguishes it from empowerment in other fields and may restrict the potential for empowerment in clinical research as well as put specific demands on the conduct of research [17, 44]. As a result, the level of patient co-determination may be limited. For example, for methodological reasons randomization might be preferable, even if alternative methods are perceived as more appropriate by the patients involved for understandable reasons. Additionally, patients may lack a full understanding of these restrictions, causing them to suggest ideas that do not comply with the logic of scientific protocols. This encounter with limitations during interactions with scientists can potentially diminish their level of empowerment.

In addition to methodological hurdles, PPI must address ethical considerations in the pursuit of empowerment. Although it is generally assumed that patient involvement does not necessitate an ethics vote, it is nonetheless crucial to discuss with potentially involved parties regarding matters such as safeguarding their privacy and potential conflict of interests, and to furnish them with comprehensive information about the involvement’s goals and methodology [45]. The framing of the involvement, and therefore the empowerment, in this manner distinguishes it from empowerment in other fields.

#### Expertise in research methods

Empowering patients in research requires providing objective support and resources to enhance their comprehension of research methods and ethics [17]. Usually, patients need assistance in navigating the complexities of research processes and methodologies [17, 46], which distinguishes empowerment in PPI from other fields. However, learning is a common aspect in any kind of empowerment. For instance, Freire’s theory of critical consciousness highlights education’s role in empowering marginalized individuals [30]. His approach centers on learners directing their own education by posing questions and emphasizes skill development over knowledge acquisition with a focus on increasing critical awareness of their circumstances.

The disparity in PPI may stem from individuals, who desire and deserve empowerment, not being the ones to decide what to learn, but from the fact that this choice is often made for them and is very factual. In terms of preparation for PPI, the learning is mostly unidirectional, whereby the researchers instruct the patients on research fundamentals [47]. However, there is a mismatch between the perception of training needs between researchers and PPI contributors (i.e. patients), both in terms of training for PPI contributors and researchers. Dudley et al. [47] found that this discrepancy leads to gaps in the support and training provided. That said, the characterization of unidirectional learning does not apply universally. For example, some PPI initiatives have employed more interactive and participatory training methods, allowing patients to engage more actively in shaping their learning experience [48].

Providing PPI support and training enables patients to acquire the necessary knowledge and skills to work alongside researchers on an equal basis, and to furnish patients with the confidence they need to challenge researchers opinions when needed [49]. Importantly, expertise in clinical research methods is not only a means of achieving empowerment but also a crucial component of enhancing the quality and relevance of research. By developing expertise, patients can contribute more meaningfully to the research process, ensuring that their perspectives and experiences are integrated in ways that improve research outcomes.

To strengthen empowerment in PPI and reduce vulnerability to co-optation by more powerful forces with different problem-solving interests, it is critical that participants have a clear understanding of the power they seek to build [34]. MacDonald’s theory of civic patienthood illustrates that socialization is central to helping patients understand their agency, role, and limitations as civic actors in PPI [34]. The design of this process can significantly impact how power and empowerment are navigated within PPI.

#### Power dynamics

Self-determination of the client is an essential aspect of empowerment practice in social work, and it is commonly believed that empowerment cannot be imposed upon anyone else [29]. In this regard, professionals are responsible for providing support and facilitation and it is crucial to minimize power differentials between all parties involved in order to foster relationships based on equality and partnership [29].

In research-based PPI, addressing power asymmetries between researchers and patients is critical. Researchers typically operate with institutions that have structures and established norms, facing constraints and pressures imposed by their institutions which can influence the extent of shared-decision making and the balance of power. Often, researchers have the final say in decisions [6]. These dynamics of institutional power can lead to challenges in achieving equal partnerships.

To navigate these constraints effectively, it is crucial to understand the extent to which patients are involved in the research process, how their roles are negotiated with researchers, and the level of their involvement in decision-making. Researchers must balance their own institutional limitations and the robustness of the research with the need to foster patient empowerment. This process can be challenging and at times frustrating. Promoting patient empowerment in clinical research impacts organizational processes, cultures and public relationships, requiring frameworks that recognize, address and integrate patient perspectives into research activities [49].

## Discussion and implications

The goal of this article is to contribute to the understanding of empowerment in PPI in clinical research by analyzing the history and development of the concept of empowerment in earlier fields. We presented an overview of the history of empowerment in the social movements of the 20th century and outlined key concepts of empowerment from Solomon, Rappaport, and Freire. Based on this, we suggested common principles of empowerment concepts. We then presented an overview of the historical development of PPI in research, that is strongly connected to the social movements’ heritage, and reflected on the relevance of empowerment in PPI. Finally, we assessed in how far empowerment in PPI mirrors the previously developed common principles of empowerment, and analyzed similarities and distinctions.

We found the heritage of social movements to inform the ethos of PPI, as principles such as promoting participation, providing people with a say in decisions that may affect their lives, appreciating diverse knowledge, fostering respectful collaborations, and confronting power structures (even at a smaller, less existential scale) are deeply embedded in PPI practices. However, we also observed considerable distinctions in contexts and objectives: Social movement-based empowerment aimed to effect systemic changes and combat inequalities. Empowerment movements typically arose from significant inequalities and were often initiated by the oppressed. While these movements laid the groundwork for later involvement in research, the empowerment objectives in PPI are more specific to the research context. Today, the involvement process is predominantly initiated by researchers seeking to incorporate patients to increase the quality and relevance of their trials.

In the practical implementation of PPI in clinical research, empowerment may often play only a minor role, irrespective of claims made to the contrary. PPI may offer ample opportunities for fostering patient empowerment, even if the primary goal is to involve patients for the enhancement of research quality or for meeting certain requirements. Nevertheless, even in trials explicitly designed to promote patient empowerment, the level of empowerment may not satisfy each individual involved. We found that these constraints are often related to researchers’ need to adhere to institutional requirements, the duration of PPI involvement, and power imbalances in relation to researchers.

Still, we feel that tentative recommendations are warranted for facilitating empowerment in clinical trials:

- Throughout the planning, execution, and dissemination of the study, close collaboration between patients and researchers is crucial. The relationship between patients and researchers should be marked by respect and mutual appreciation [4]. Both parties should value all perspectives and prioritize inclusivity in decision-making processes. MacDonalds’ model of civic patienthood offers valuable insights for strengthening patients’ voice and the power dynamics in PPI [34].
- As defined by Salomon, the success of empowerment depends on “the extent to which the service delivery system functions as either an obstacle course or an opportunity system” [26]. In the case of PPI, the study and patient involvement should be designed in such a way that patients fully understand the process and its realistic limitations. It is essential to make the research accessible and transparent, with clear communication about what it can and cannot promise. Acknowledging the limitations of clinical research as a vehicle for empowerment respects patients’ capacity to understand these limitations and helps manage their expectations, fostering a more honest and trustful relationship between researchers and patients [16, 17].
- Prior to and throughout their collaboration, patients and researchers should engage in discussions about their shared objectives, expectations, and experiences [16]. These should include notions of empowerment and empowerment should be an aspect that guides the involvement.
- To promote collaborative equality, patients may participate in training sessions prior to or at the beginning of their involvement. These sessions should offer a comprehensive understanding of clinical research and enhance their perspective as patients, empowering them to challenge researchers when necessary [3]. In the spirit of peer support and collective action [30], patients themselves may offer these training sessions for the benefit of their fellow patients, thereby reducing power imbalances in the learning environment.
- Researchers ought to engage in training sessions for PPI [50], including instructions on how to foster empowerment.
- Patients collaborating with researchers should be accompanied and supported as needed by a person who feels responsible and plays a role similar to that of a social worker in other contexts [29]. Despite time constraints in PPI, there should be opportunities for patients to share and analyze experiences, provide mutual support, and collaborate during the course of the clinical trial [18, 30].
- At the end of the participation, there should be a closing session where, among other things, the participation is reflected upon and its added value is highlighted [17]. This includes not only aspects that have changed the quality of the study, but also, for example, changes and developments at the personal level of patients and researchers. Patients who wish to continue their involvement should have opportunities to do so.

This list presents several ways for promoting empowerment within the context of PPI. It is not conclusive but rather intended to be extended and elaborated upon in further examinations of the subject. However, defining empowerment is a complex undertaking, and one may select different criteria or aspects that may lead to alternative approaches to promoting it.

## Conclusions

The primary objective of clinical research is not to empower patients but to generate scientific knowledge that can improve healthcare outcomes. However, with the increasing call for involving patients in research, the concept of empowerment has become an associated goal. Our investigation sought to unpack what empowerment might mean within the context of PPI in clinical research.

Given the absence of a consensus on what empowerment in this context entails, we turned to the history and foundational concepts of empowerment from various social movements to illuminate its potential meanings and implications. We found both similarities and differences between empowerment in PPI and earlier empowerment concepts. While PPI reflects principles such as participation, challenging power structures, and valuing diverse perspectives, the empowerment it offers is often constrained by the specific context of clinical research.

Some limitations to empowerment in PPI are intrinsic to the research context itself, such as the need to adhere to rigorous scientific standards. However, other limitations are less evident and may, in fact, undermine the empowerment of patients. These include institutional power dynamics, limited opportunities for genuine decision-making, and inadequate support for patients to navigate the complexities of research processes.

To address these challenges, it is crucial for those involved in PPI to be explicit about what they mean by empowerment and to consider whether and how it is valued in their research endeavors. Transparency regarding both external and internal limitations is essential. This includes an explicit exchange between researchers and patients about the realistic scope and potential of patients’ involvement, as well as ongoing reflection and dialogue about how empowerment can be fostered and sustained within the research process. By doing so, PPI can move closer to fulfilling its promise of genuinely empowering patients, rather than merely using the term as a rhetorical tool.

## Data Availability

No datasets were generated or analysed during the current study.

## Abbreviations

PPI: Patient and Public Involvement
